# The PAXgene^®^ Tissue System Preserves Phosphoproteins in Human Tissue Specimens and Enables Comprehensive Protein Biomarker Research

**DOI:** 10.1371/journal.pone.0060638

**Published:** 2013-03-29

**Authors:** Sibylle Gündisch, Christina Schott, Claudia Wolff, Kai Tran, Christian Beese, Christian Viertler, Kurt Zatloukal, Karl-Friedrich Becker

**Affiliations:** 1 Institute of Pathology, Technische Universität München, Munich, Germany; 2 Institute of Pathology, Medical University of Graz, Graz, Austria; 3 The SPIDIA Consortium, Qiagen Research and Development, Hilden, Germany; Complutense University, Spain

## Abstract

Precise quantitation of protein biomarkers in clinical tissue specimens is a prerequisite for accurate and effective diagnosis, prognosis, and personalized medicine. Although progress is being made, protein analysis from formalin-fixed and paraffin-embedded tissues is still challenging. In previous reports, we showed that the novel formalin-free tissue preservation technology, the PAXgene Tissue System, allows the extraction of intact and immunoreactive proteins from PAXgene-fixed and paraffin-embedded (PFPE) tissues. In the current study, we focused on the analysis of phosphoproteins and the applicability of two-dimensional gel electrophoresis (2D-PAGE) and enzyme-linked immunosorbent assay (ELISA) to the analysis of a variety of malignant and non-malignant human tissues. Using western blot analysis, we found that phosphoproteins are quantitatively preserved in PFPE tissues, and signal intensities are comparable to that in paired, frozen tissues. Furthermore, proteins extracted from PFPE samples are suitable for 2D-PAGE and can be quantified by ELISA specific for denatured proteins. In summary, the PAXgene Tissue System reliably preserves phosphoproteins in human tissue samples, even after prolonged fixation or stabilization times, and is compatible with methods for protein analysis such as 2D-PAGE and ELISA. We conclude that the PAXgene Tissue System has the potential to serve as a versatile tissue fixative for modern pathology.

## Introduction

For accurate diagnosis of diseases such as cancer, histopathological analysis is still the gold standard. In recent years, however, molecular diagnostics and the elucidation of the mechanistic pathways of malignancy have been incorporated into routine pathology for the purpose of stratifying cancer patients for the most effective individualized therapy. Crucial to these personalized approaches to therapy and for biomarker discovery programs is the analysis of high quality clinical tissue specimens which are suitable for conventional histopathological diagnosis as well as accurate detection and quantification of nucleic acids and proteins. Tissue preservation plays a key role in this context.

The two most commonly used preservation methods are snap freezing or formalin fixation and paraffin-embedding (FFPE) of surgical specimens. Both methods have advantages and disadvantages which have been intensively discussed in the last few years [1], [2], [3]. For decades, FFPE tissues have been the only available material for histological evaluation, but formalin is carcinogenic [4] and cross-links biomolecules, a property of this fixative which is still problematic for many downstream molecular analyses [1], [5], [6], [7]. Although, improvements of the standard formalin fixation process, such as formalin fixation at low temperature, demonstrate a better preservation of the integrity of nucleic acids [8]. This approach improves the preservation of the molecular content of FFPE tissue but, nevertheless, the level of nucleic acid quality obtained from cryopreserved tissue samples is not reached. Another recently published study focused on the preservation of phosphoproteins and tissue morphology by a novel biomarker and histology preservative (BHP) [9]. The novel formalin-free fixation technology PAXgene Tissue System has previously been shown to simultaneously preserve tissue morphology and antigenicity, as well as nucleic acids and proteins [10], [11], [12]. More important is the fact that PAXgene-fixed and paraffin-embedded tissues (PFPE) provide nucleic acid and protein quantity and quality similar to that found in cryopreserved tissue samples.

In a previous publication, we presented the first data on the evaluation of PFPE tissues for proteomic analysis [10]. The present study goes well beyond these early analyses and is aimed at studying in more detail the capability of the PAXgene Tissue System to preserve phosphoproteins in clinical tissue specimens, including cancer tissues. Phosphoproteins are of critical importance in the analysis of tumor specimens as the inhibition of kinases has become a major focus of anti-cancer drug development [13]. It is imperative, therefore, that before a new tissue preservation tool is adopted for routine clinical use, it must be shown that post-translational modifications such as phosphorylation of proteins are not altered by the preservation method used. In this study, we systematically compared phosphoprotein levels extracted from human malignant and non-malignant cryopreserved, PFPE and FFPE tissue samples. We also evaluated the applicability of proteins extracted from PFPE tissues for two-dimensional gel electrophoresis (2D-PAGE) and enzyme-linked immunosorbent assays (ELISA). For retrospective studies as well as biobanking of tumor tissue, another critical factor is the stability of proteins and phosphoproteins within tissues preserved by different means and for various storage times at different temperatures. The present study also addresses this important issue.

## Materials and Methods

### Human tissue samples

Non-malignant (stomach, oesophagus, colon, duodenum, ovary, uterus, breast, prostate, bladder, spleen, pancreas, gall bladder, muscle, skin, tongue, salivary gland, thyroid gland, lung and cystadenoma) and malignant (ovarian, breast and prostate cancer) human tissue specimens were collected during routine surgical procedures at the Institute of Pathology, Klinikum Rechts der Isar of the Technische Universität München, Munich, Germany. Non-malignant (liver, stomach, spleen) and malignant (leiomyosarcoma) human tissue specimens were collected at the Institute of Pathology, Medical University of Graz, Austria. In total, we obtained 20 different non-malignant and 4 different malignant tissue types for this study. Each specimen was equally divided and either snap frozen in liquid nitrogen (LN_2_), fixed in 3.5–3.7% neutral buffered formaldehyde or fixed with PAXgene Tissue Fix (PreAnalytix GmbH, Hombrechtikon, Switzerland) for various time points at ambient temperature. PAXgene-fixed samples were transferred into PAXgene Tissue Stabilizer at different time points to stop fixation and store the tissue samples for subsequent processing. Using a standard protocol, PAXgene treated and formalin-fixed tissues were dehydrated and embedded in low-melting temperature paraffin in respectively dedicated devices to avoid cross-contamination of the two fixatives. The protocol consists of stepwise dehydration in 70%, 80%, 90%, and 99% ethanol (two baths), followed by isopropanol (two baths) and xylene (two baths). PFPE and FFPE blocks were stored 4°C or room temperature in the dark. All patients signed written informed consent, and the study was approved by the Ethics Committee of the Klinikum Rechts der Isar of the Technische Universität München, Munich, Germany (reference number 2336/09) and by the Ethics Committee of the Medical University of Graz (reference number 20-066).

### Protein extraction

Proteins from cryosections were extracted by homogenization in extraction buffer EXB Plus [14] (Qiagen GmbH, Hilden, Germany), supplemented with protease (Complete Protease Inhibitor Cocktail), phosphatase (PhosSTOP Phosphatase Inhibitor Cocktail, both Roche Applied Science, Mannheim, Germany) and kinase inhibitors (staurosporine and genistein, Sigma-Aldrich Chemie GmbH, Munich, Germany), in a pre-cooled homogenizer for 2–4 min at 50 Hz (Tissue Lyser LT, Qiagen GmbH, Hilden, Germany) and further disruption by sonification on ice (Branson Digital Sonifier, Heinemann, Schwäbisch Gmünd, Germany). After centrifugation at 4°C for 5 min at 14.000 x g, the supernatant was stored at –20°C for short-term (< four weeks) and at -80°C for long-term (> four weeks) storage. Proteins from PFPE tissues were extracted as described previously [10]. Proteins from FFPE tissues were also extracted as described previously according to the Qproteome FFPE Tissue Kit Handbook (Qiagen GmbH, Hilden, Germany) [15]. Protein concentration was determined using the Bradford assay and protein yield was calculated as described previously [10].

### Western blot analysis

Equal amounts of protein lysates were separated by one-dimensional SDS-PAGE and blotted onto nitrocellulose membranes. Protein detection was performed by incubation with respective antibodies (**Table S1**), and proteins were visualized using the ECL Plus Western blot detection reagent (Amersham/GE Healthcare Europe GmbH, Freiburg, Germany). For protein visualization by a fluorescence detector (FluorChemSP, Alpha Innotech, Biozym, Hessisch Oldendorf, Germany) the ChemiGlow reagent (Cell Biosciences Ins., CA, USA) was applied.

### Two-dimensional SDS-PAGE

Proteins from cryopreserved samples were extracted in 2D buffer (30 mM Tris-HCl pH 8.8, 7 M urea, 2 M thiourea, 4% CHAPS, 75 mM DTT) supplemented with protease inhibitor (Complete Protease Inhibitor Cocktail, Roche Applied Science, Mannheim, Germany) in a pre-cooled homogenizer for 4 min at 50 Hz (Tissue Lyser LT, Qiagen GmbH, Hilden, Germany). PFPE samples were lysed in 2D buffer supplemented with protease inhibitor on ice for 30 min. Proteins from FFPE tissues were extracted in EXB Plus buffer supplemented with protease inhibitor as described above and subsequently precipitated by the addition of three volumes of acetone. FFPE protein lysate (100 µl) was mixed with 300 µl of ice cold acetone followed by incubation on ice for 4 h or over night. After centrifugation at 4°C for 30 min at 14.000 x g, the supernatant was discarded and the air-dried protein pellets were resuspended in 2D buffer. For overnight in-gel rehydration into IPG strips (18 cm, pH 3–10 NL) (BioRad, Hercules, USA), each lysate containing 150 µg protein was dissolved in 315 µl rehydration buffer (7 M urea, 2 M thiourea, 0.4% DTT, 4% CHAPS, 0.5% Bio-Lyte 3/10 ampholytes (BioRad, Hercules, USA), 0.001% bromphenol blue). Focusing was performed using the IEF100 Isoelectric Focusing Unit (Hofer, Holliston, USA) to a total of 55000 Vh (2 h at constant 250 V, 1 h gradient up to 500 V, 1 h constant at 500 V, 1 h gradient up to 1250 V, 1 h constant at 1250 V, 2 h gradient up to 8000 V, 4 h constant at 8000 V). After isoelectric focusing, strips were equilibrated in 6 M urea, 2% SDS, 30% glycerol, 50 mM Tris-HCl (pH 8.8) supplemented with 1% DTT for 10 min, and then with 2.5% iodoacetamide for additional 10 min. For second-dimension electrophoresis, IPG strips were subjected to SDS-PAGE using 12% polyacrylamide gels (26×20 cm) in the Ettan DALTsix system (GE Healthcare, Buckinghamshire, UK) at 1 W per gel, until the dye front reached the end of the gel. The gels were then fixed in 50% methanol, 10% acetic acid for at least 30 min and the proteins were stained using the fixation solution supplemented with 0.25% Coomassie Blue R-250 for 1 h. Destaining was performed for 4-24 h, in 5% methanol, 7.5% acetic acid.

### ELISA

Pilot studies revealed that proteins from PFPE tissues extracted according to our standard protocol [10] were not compatible with subsequent ELISA assay due to high SDS concentrations in the extraction buffer interfering with the antibodies in the assays. We therefore used buffers recommended by the manufacturers of the ELISA kits for protein extraction.

For the Erk-1/2 (total) and phospho-Erk-1/2 [pTpY185/187] Human ELISA Kits (Invitrogen Corp., CA, USA), proteins were extracted with the Denaturing Cell Extraction Buffer (DCEB, Invitrogen Corp., CA, USA) supplemented with protease inhibitors. For the Akt (total) Human ELISA Kit (Invitrogen Corp., CA, USA), proteins were extracted from PFPE and frozen tissues with the Cell Extraction Buffer (CEB, Invitrogen Corp., CA, USA). As CEB produced lower than expected protein yields with PFPE tissue, tris-buffered saline, pH 8.5, 1% Triton-X-100 (American Diagnostica, CT, USA), supplemented with protease inhibitors was used to extract proteins from PFPE and frozen tissues for the Akt ELISA. The ELISA assays were performed with protein lysates from three non-malignant (stomach, duodenum and uterus) and three malignant (breast metastasis, ovarian and prostate cancer) tissue types following the manufacturer’s instructions.

## Results

### The PAXgene Tissue System preserves proteins and phosphoproteins in clinical tissue specimens

To gain insights into the capability of the PAXgene Tissue System to preserve the phosphoproteome, in the current study we investigated different human malignant (n = 3) and non-malignant (n = 17) tissues with particular emphasis on phosphoproteins. Proteins of corresponding cryopreserved, PFPE and FFPE tissues were extracted as described in the Methods section and analyzed by western blot with 13 antibodies, 11 of which were directed against phosphorylated epitopes.

**Figure 1:**
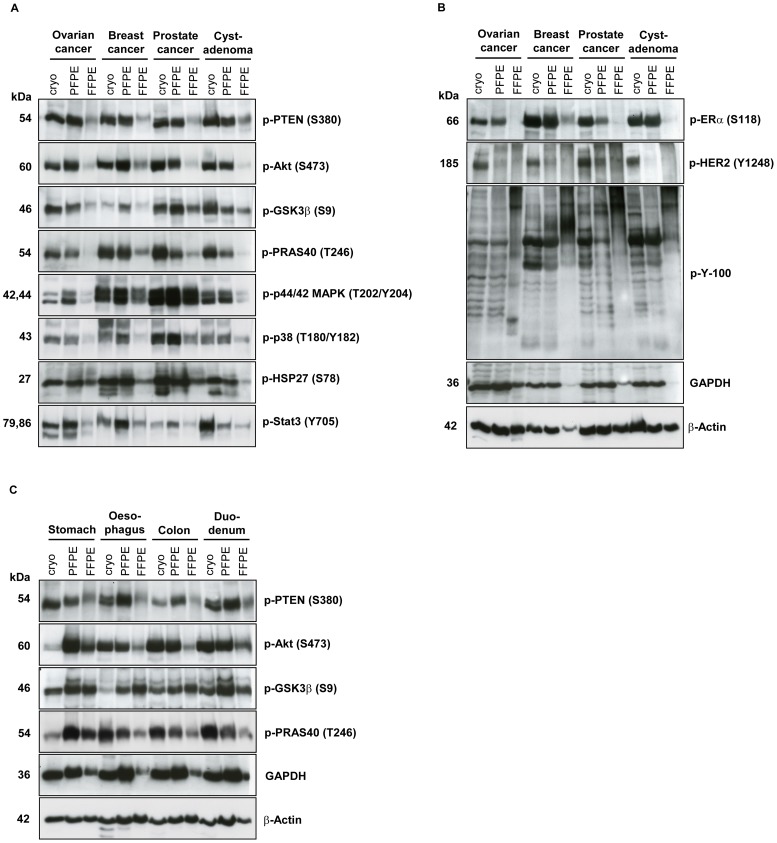
The PAXgene Tissue System preserves phosphoproteins in human clinical tissue specimens. (**A, B**) Three human malignant (ovarian, breast and prostate cancer), one pre-malignant (cystadenoma) and (**C**) four non-malignant (stomach, oesophagus, colon, duodenum) tissue specimens were each divided into three samples and either cryopreserved (cryo), fixed and stabilized in the PAXgene Tissue reagents and paraffin-embedded (PFPE) or fixed in formalin and paraffin-embedded (FFPE). Proteins were extracted with respective protocols (See Protein Extraction, Experimental Section) and 15 µg protein of each was separated by SDS-PAGE. Western blot analysis was performed using indicated antibodies.

The results (**Figure 1** **and Figure S1**) clearly demonstrate that phosphoproteins are preserved in both malignant and non-malignant PFPE human tissue specimens. The signal intensities obtained from cryopreserved and PFPE samples were similar for most of the investigated proteins, with the exception of phospho-PRAS40 in skin, and phospho-GSK3, phospho-Akt, and phospho-PTEN in spleen, for example (**Figure S1 B**). For the latter three phosphoproteins, higher signals were obtained with PFPE than with frozen tissue. Conversely, phosphoproteins from FFPE tissues almost always produced weaker signals than those of cryopreserved or PFPE tissues and would have needed much longer exposure times to reach equal signal intensities. The pattern of phosphorylated tyrosine residues, as detected by the phospho-tyrosine antibody (p-Y-100), was also similar for cryopreserved and PFPE tissues, whereas, signals from FFPE tissues were almost undetectable during the exposure time used, especially in the lower molecular weight fraction (**Figure 1B**). Phospho-HER2 was the only phosphoprotein for which we could detect only very weak signals in PFPE samples compared to those obtained from cryopreserved tissue and even no signals from FFPE tissues (**Figure 1B**).

### Proteins extracted from PAXgene fixed and stabilized clinical tissue specimens are suitable for two-dimensional gel electrophoresis

**Figure 2:**
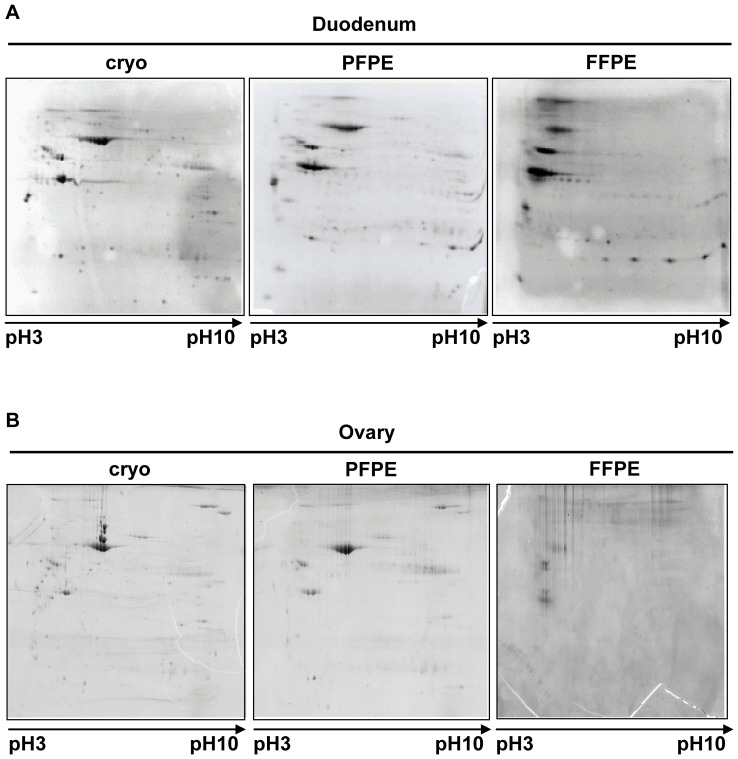
Proteins extracted from PFPE tissues are suitable for two-dimensional gel electrophoresis. Two human non-malignant (duodenum, ovary) tissue specimens were each divided into three samples and either cryopreserved (cryo), fixed and stabilized in the PAXgene Tissue reagents and paraffin-embedded (PFPE) or fixed in formalin and paraffin-embedded (FFPE). Proteins were extracted with respective protocols (See Two-dimensional SDS-PAGE, Experimental Section) and 150 µg protein of each was separated by two-dimensional SDS-PAGE. The isoelectric focusing was conducted in a range between pH 3–pH 10.

Proteins from five human non-malignant tissue types (duodenum, colon, stomach, ovary, and breast) were extracted and analyzed by 2D-PAGE. Representative examples are shown in **Figure 2** and **Figure S2**. The results demonstrate that the spot pattern obtained from cryopreserved and PFPE samples were similar. Lysates from FFPE samples, however, appeared not to separate in both dimensions and therefore a satisfying separation of the FFPE lysates could not be achieved (**Figure 2** **and Figure S2**). The reproducibility of the system was confirmed by replicate analyses of the duodenum sample (**Figure S2 A**). Slight difference in spot intensities from PFPE samples compared to the respective cryopreserved samples was detectable in stomach and breast tissue, but the overall quality of results obtained with PFPE lysates on 2D-PAGE gels was still superior to those of FFPE samples (**Figure S2 C and D**).

These results demonstrate that proteins extracted from PFPE samples are suitable for 2D-PAGE. Slight differences in spot intensity were detectable for only two investigated tissue types which could also occur due to inter-gel variability. In contrast, proteins extracted from FFPE samples couldn’t be separated adequately on 2D-PAGE gels and the results from these lysates, therefore, were hardly comparable to results from either cryopreserved of PFPE samples.

### Proteins extracted from PAXgene fixed and stabilized clinical tissue specimens are only suitable for enzyme-linked immunosorbent assays specific for denatured proteins

Typically, enzyme-linked immunosorbent assays (ELISA) enable the detection of antigens in a liquid sample and are important tools for routine clinical diagnostics. To evaluate the applicability of PFPE samples for ELISA, we performed sandwich ELISA assays with capture antibodies detecting either denatured or non-denatured proteins. We investigated malignant and non-malignant tissues with three different commercial ELISA kits, one of which was specific for non-denatured proteins (Akt/Proteinkinase B) while the other two were meant to work with denatured proteins (extracellular signal-regulated kinase-1/2 (Erk-1/2) and phospho-Erk-1/2).

**Figure 3:**
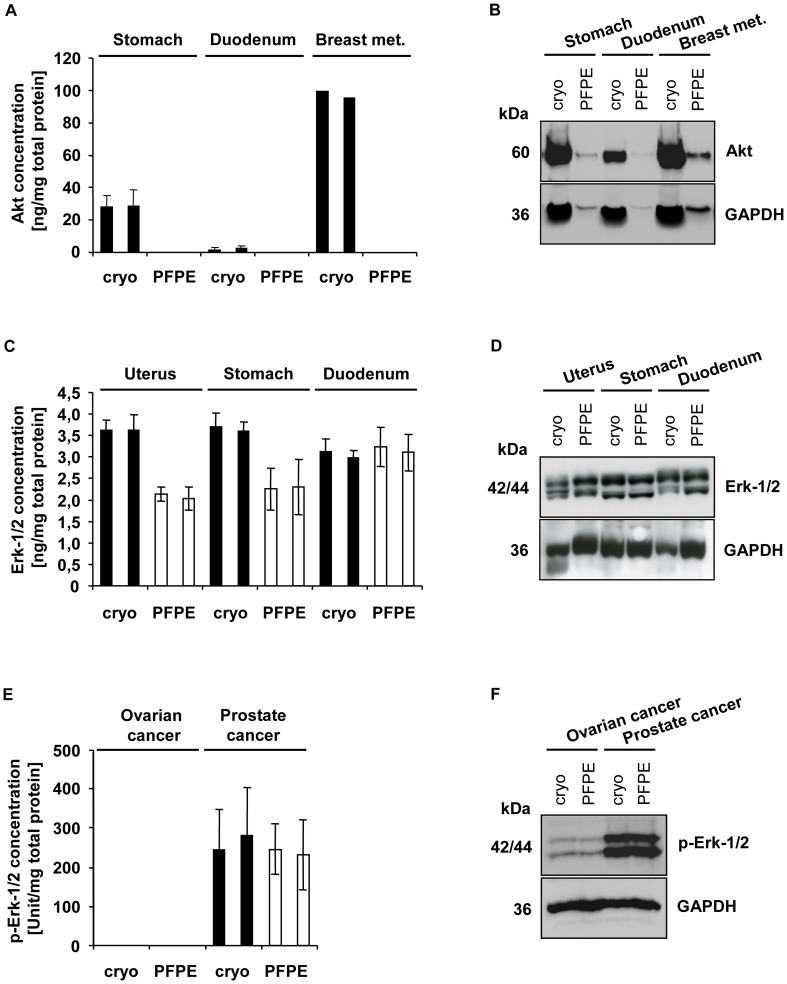
Proteins extracted from PFPE tissues are suitable for ELISAs with antibodies directed against denatured proteins. Three human malignant (breast cancer metastasis, ovarian and prostate cancer) and three non-malignant (stomach, duodenum, uterus) tissue specimens were each divided into two samples and either cryopreserved (cryo) or fixed and stabilized in the PAXgene Tissue reagents and paraffin-embedded (PFPE). Proteins were extracted with respective protocols (See ELISA, Experimental Section) using denaturing or non-denaturing extraction buffer. Results are always depicted in duplicates for each lysate. (**A**) An ELISA for non-denatured Akt (n = 2 for stomach and duodenum samples. (**C**) An ELISA for denatured Erk-1/2 (n = 2). (**E**) An ELISA for denatured phospho-Erk-1/2 (n = 3). Western blot analysis of (**B)** 12.5 µg or (**D, F**) 25 µg protein of each lysate separated by SDS-PAGE and performed using indicated antibodies.

We performed protein extraction with the non-denaturing cell extraction buffer (CEB) recommended for the Akt ELISA, but we were not successful in extracting proteins from PFPE samples using this method. Protein extraction efficiency improved with the use of a buffer containing Triton-X-100 which effected a mild, non-denaturing extraction. The extraction efficiency from PFPE samples with this buffer was better than with CEB, but the protein yield was still about 70% lower than the protein yield from cryopreserved samples (data not shown). We performed an Akt ELISA with these protein lysates but were not successful in detecting any signals above the background signal (**Figure 3A**). To validate the results, we performed western blot analysis with the same lysates, which confirmed very weak signals for Akt (**Figure 3B**). GAPDH was also very weak in the PFPE lysates obtained with the mild non-denaturing buffer indicating poor extraction efficiency.

Protein extraction with the denaturing cell extraction buffer (DCEB) recommended for the Erk-1/2 and phospho-Erk-1/2 ELISA, yielded high protein concentrations from both cryopreserved and PFPE samples. The Erk-1/2 ELISA showed reproducibly high signal intensities for all three tissue types and for both preservation methods (**Figure 3C**). For the duodenum samples the results obtained were similar for both preservation methods, whereas the overall signal intensities from stomach and uterus PFPE samples were on average about 40% lower compared to paired croypreserved samples. Western blot analysis, however, did not reveal the same difference in signal intensity between cryopreserved and PFPE stomach and uterus tissue (**Figure 3D**). The phospho-Erk-1/2 ELISA results correlated well to the western blot analysis (**Figure 3E and F**). For prostate cancer samples, no difference in signal intensity could be detected between both preservation methods, and phospho-Erk-1/2 could not be detected in or could be detected at very low levels in cryopreserved and PFPE ovarian cancer tissues by ELISA or western blot analysis (**Figure 3E and F**).

### Impact of prolonged fixation or stabilization time on protein yield and quality

In a routine clinical setting tissue specimens could be fixed for more than 24 hours due to different circumstances, such as weekend or holidays. It is therefore important to evaluate whether there is a negative impact on protein yield or quality of a prolonged incubation time in either PAXgene Tissue Fix or Stabilizer.

With this question in mind, we obtained a human pancreas tissue specimen which was aliquoted and the aliquots fixed with PAXgene Tissue Fix for different periods of time up to 120 hours, before transfer into PAXgene Tissue Stabilizer. These samples were processed and embedded in paraffin, and proteins were extracted. The protein concentration was determined to calculate the total protein yield, and the quality of extracted proteins was assessed by western blot analysis.

**Figure 4:**
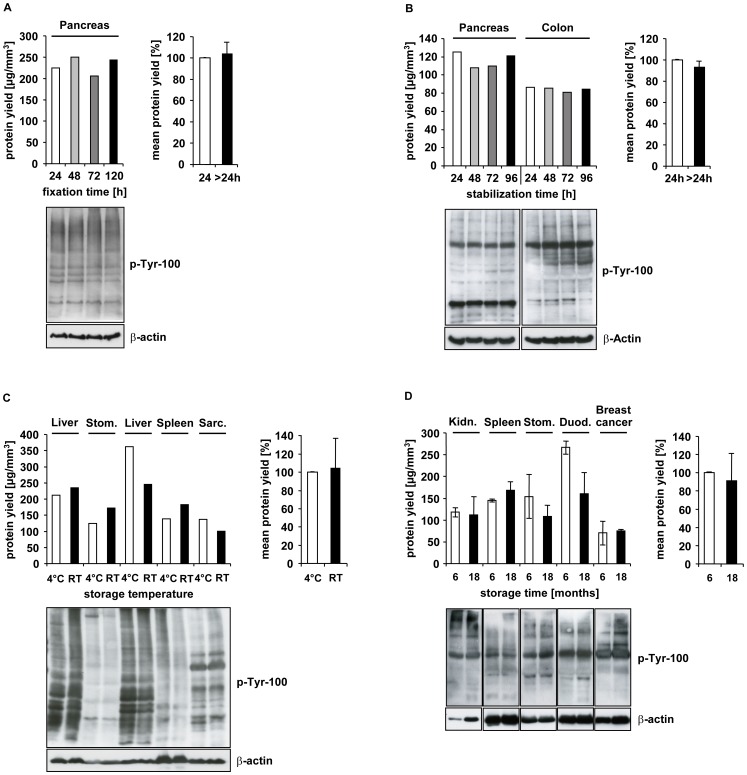
Impact of fixation or stabilization time, storage temperature and time on proteins from PFPE tissues. **(A)** A human pancreas tissue specimen was divided into four samples and fixed with the PAXgene Tissue Fix reagent for 24, 48, 72 or 120 hours and transferred to PAXgene Stabilizer reagent for 24 hours before paraffin-embedding. (**B**) Human pancreas and colon tissue specimens were each divided into four samples and fixed with the PAXgene Tissue Fix reagent for 24 hours and transferred to PAXgene Stabilizer reagent for 24, 48, 72 or 96 hours before paraffin-embedding. (**C**) Four human non-malignant (liver (n = 2), stomach, spleen) and one malignant (leiomyosarcoma) tissue specimens were each divided into two samples and fixed (24 hours) and stabilized (24–72 hours) with the PAXgene Tissue System before paraffin-embedding. The storage temperature was either 4°C or room temperature (RT). (**D**) Eight human non-malignant (kidney, spleen, stomach, duodenum, all in duplicates) and two malignant (breast cancer) tissue specimens were aliquoted and fixed (3 or 24 hours) and stabilized (24 hours) with the PAXgene Tissue System before paraffin-embedding. Protein extraction and determination of protein concentration was either performed after six months of storage (white bars) or after additional 12 months of storage (black bars) always under the same conditions (e.g. same number of tissue sections and same volume of extraction buffer). (**A, B, C, D**) Proteins were extracted and the protein concentration was determined to calculate the total protein yield which is depicted. (**A, B, D**) 20 µg or (**C**) 50 µg protein was separated by SDS-PAGE and western blot analysis was performed using indicated antibodies.

The results show that a prolonged fixation time in PAXgene Tissue Fix up to 120 hours had no negative effect on subsequent protein extraction, either on total protein yield or on protein quality as determined by an antibody directed against all phosphorylated tyrosine residues (**Figure 4A**). The mean protein yield of those samples fixed for more than 24 hours, compared to 24 hours, was approximately 104%. Western blot analysis revealed that the proteins were immunoreactive and not degraded, regardless of the fixation time. These results could also be duplicated in liver samples which were fixed in PAXgene Tissue Fix for different time periods between 4 h – 120 h (data not shown).

To investigate the impact of prolonged stabilization time, human pancreas and colon tissue specimens were aliquoted and the aliquots fixed with PAXgene Tissue Fix for 24 hours and transferred into PAXgene Tissue Stabilizer for different time periods up to 96 hours prior to processing and embedding in paraffin. Protein yield and quality were evaluated as described above.

The data suggest that a prolonged stabilization time for up to 96 hours has no negative effect on subsequent protein extraction, either on total protein yield or on quality (**Figure 4B**). A slight decrease in protein yield over the storage time was observed in the pancreatic tissue specimens, but no differences could be detected in the colon samples over the same period. The mean protein yield of specimens stabilized for more than 24 hours compared to the yield obtained at 24 hours stabilization time was approximately 93%. Western blot analysis, however, showed that these proteins are immunoreactive and not degraded, irrespective of the stabilization time.

### Impact of storage temperature and time on protein yield and quality

In routine clinical archives FFPE samples are stored at room temperature (RT) and it is known that protein recovery decreases with increasing storage time [16]. It is therefore important to evaluate the impact of storage temperature and time on protein yield and quality in PFPE samples.

For this purpose, different paired PFPE human tissue samples were either stored at 4°C or at RT respectively for a minimum of one year. Protein yield and quality were evaluated as described above.

For two out of five tissue samples (liver, leiomyosarcoma) we could detect a slight decrease in protein yield of approximately 30% when PFPE samples were stored at RT as compared to 4°C (**Figure 4C**), however, the mean protein yield over all five samples analyzed showed no significant differences. Furthermore, in another liver sample used for this evaluation, no difference in protein yield was detectable as a function of storage temperature. Western blot analysis revealed that the pattern of phosphorylated proteins was different for the various tissue types, but the pattern was independent of storage temperature.

To evaluate the impact of storage time on protein stability, we analyzed PFPE blocks, which were stored for 18 months at 4°C. The initial storage age of the PFPE tissues prior to the first protein extraction was 6 months and protein extraction was repeated in exactly the same manner 12 months after the first extraction had been performed. Protein yield and quality were evaluated as described above. The results clearly show that storing PFPE tissue for more than one year at 4°C has no major negative effect on protein yield and quality which was also confirmed by western blotting (**Figure 4D**). On average, the mean protein yield of PFPE samples stored for more than one year was slightly reduced by about 9% compared to the initial protein extraction. Long-term studies concerning the impact of storage time and conditions are ongoing.

## Discussion

In recent years, several attempts have been made to replace formalin by other preservatives in order to improve the quality of tissue-based proteomic and molecular studies [9], [17], [18], [19], [20]. Unfortunately, completely satisfactory results have not yet been achieved with these preservation techniques. In previous publications we have demonstrated that the PAXgene Tissue System has potential as a novel tissue preservation technology for simultaneous preservation of tissue morphology and antigenicity, as well as the integrity of nucleic acids and proteins [10], [11], [12]. However, the PAXgene tissue stabilization system is not intended to be a general formaldehyde replacement; instead, it may facilitate analysis of diseased tissues for which collection of frozen material for molecular analyses is difficult or not feasible. Such studies will start in the near future. In addition, PFPE tissue collections may be useful for biobanks where morphology and molecular analysis are to be performed from the same piece of tissue. In the present study, we focused on the preservation and stability of phosphoproteins in PFPE tissue and the suitability of proteins extracted from PFPE samples for 2D-PAGE and ELISA. Moreover, we addressed clinically relevant situations about fixation time, stabilisation time, storage time, and storage temperature because in a routine clinical setting these parameters may vary from sample to sample.

First, we were interested in whether the PAXgene Tissue System had the potential to preserve phosphoproteins in clinical tissue samples. For this purpose, we evaluated three malignant, one pre-malignant, and 16 non-malignant human tissues. The extraction of proteins and phosphoproteins from PFPE tissues is much simpler compared to other methodologies, e.g. FFPE-FASP [21], and does not require urea and a filtration step [10]. Using 13 antibodies, 11 of which were directed against phosphorylated epitopes, we compared western blot signal intensities from cryopreserved, PFPE and FFPE samples for all tissues. The detected signal intensities from cryopreserved and PFPE samples were comparable for all 11 phosphoproteins analyzed except phospho-HER2, whereas signals obtained from FFPE tissues were, by comparison, much weaker. Unexpectedly, the signals for phospho-HER2 were always weaker in PFPE samples compared to cryopreserved samples. The reason for this finding is unclear and needs further investigations with other high molecular weight proteins or other membrane proteins. For FFPE samples, longer exposure times were needed to detect high molecular weight proteins. A possible explanation could be that incomplete reversal of protein crosslinks leave high molecular weight complexes in the protein lysates which cannot migrate properly into the poylacrylamide gel and thus are retained at the top of the gels.

In some cases, slight differences between signal intensities from cryopreserved and PFPE tissues were detected for phosphoproteins other than phospho-HER2. For example, the signals for phospho-PRAS40 detected in prostate cancer and cystadenoma samples were weaker for PFPE compared to cryopreserved samples. On the other hand, for example in stomach samples, the signals for phospho-Akt and phospho-PRAS40 detected from cryopreserved samples were weaker than those for PFPE samples.

Our data indicate that phosphoprotein signal discrepancies between the PFPE and cryopreserved tissue may have resulted from tissue-specific problems such as tissue heterogeneity rather than a deficiency in the PAXgene Tissue fixation and stabilization technology. This hypothesis is supported by results from a current study in which we are performing a comprehensive comparative analysis of the phosphoproteome of cryopreserved and PFPE rat liver samples using quantitative tandem mass spectrometry. Data obtained from more than 3000 detected phosphosites are in line with the results described in the present study, namely that the PAXgene Tissue system quantitatively preserves the phosphoproteome of tissue samples and enables even mass spectrometric based analysis (Gündisch, et al., manuscript in preparation). In future clinical settings phoshoproteins preserved in PAXgene fixed tissues may serve as companion diagnostics to select patients for drugs targeting activated signalling pathways (e.g. the PI3K/AKT/mTOR pathway).

In order to study the suitability of protein extracts from PFPE samples for two-dimensional SDS-PAGE (2D-PAGE), we investigated five human non-malignant tissues. The results demonstrated that the spot pattern as well as the spot intensity from cryopreserved and PFPE samples were similar, while in contrast, the spots from FFPE tissue displayed a markedly different distribution pattern and generally weaker intensity. Addis, *et al.* recently showed that it is possible to obtain matching 2D protein profiles from FFPE and frozen tissues [22], [23]. These researchers successfully performed two-dimensional differential gel electrophoresis (2D-DIGE). Through the reduction of gel-to-gel variability, this technique is considered to be the most refined technology for performing gel-based differential proteomic investigations [24], [25].

Of all tissues tested, differences in spot intensities between PFPE and cryopreserved samples were detected only in stomach and breast tissues. The spots detected from PFPE samples were weaker than those for cryopreserved tissue. This may have been due to gel-to-gel or technical variability. Nevertheless, the overall gel quality of PFPE samples was still superior to the FFPE samples and, replicate analysis of a duodenum sample showed that the results were highly reproducible with regard to both spot distribution and intensity for PFPE samples. Thus, we conclude that preservation of tissue samples with the PAXgene Tissue System has no negative impact on spot distribution and signal intensities in 2D-PAGE analysis, and proteins extracted from PFPE tissues are suitable for 2D-PAGE.

Another important technique for tissue proteomics is the high-throughput, multianalyte system enzyme-linked immunosorbent assay (ELISA). While important for e.g. cancer biomarker validation studies high inter-array and day-to-day variability may occur [26], [27]. We investigated whether proteins extracted from PFPE blocks are generally suitable for ELISA. We were successful in performing ELISA with primary antibodies directed against denatured proteins in malignant and non-malignant tissue samples. The signal intensities from PFPE samples were in some assays weaker compared to cryopreserved samples, but in general , the results were reproducible for different tissue types and correlated to the western blot analysis which we performed in parallel. Problems were observed for ELISA with primary antibody directed against non-denatured proteins. Protein extraction was inefficient because mild, non-denaturing buffers with low detergent (Triton-X-100) concentrations had to be used. The total protein yield obtained from PFPE samples, therefore, was about 70% lower than that of cryopreserved samples. This decrease in protein yield had been detected by previous western blot analysis. Nevertheless, we performed the ELISA with these suboptimal lysates, but were not successful in detecting signals from PFPE samples above background.

These results indicate that proteins extracted from PFPE samples are suitable for ELISA assays specific for denatured proteins. At this time, we cannot determine whether the negative results we obtained with the ELISAs specific for non-denatured proteins were due to the poor extraction efficiency from PFPE tissue or to the presence of denaturing effects inherent in the PAXgene Tissue System fixation/stabilization process.

These issues need to be investigated in future studies.

To evaluate whether a new tissue fixative is suitable for use in a routine clinical setting, it is important to investigate the impact of several critical parameters, such as prolonged fixation or stabilization times as well as storage times and temperatures on protein quantity and quality. For FFPE tissues, it is known that prolonged fixation and storage times do have a negative impact on protein yield [14], and that loss of antigenicity can occur in archived FFPE tissue sections [14], [17]. The results in the present study clearly demonstrate that neither a prolonged fixation time up to 120 hours nor a prolonged stabilization time up to 96 hours has a major negative impact on total protein yield and quality. Additionally, the data suggest that neither storage temperature nor time have a major negative impact on protein quantity and quality in PFPE samples. The proteins extracted from tissues stored at various temperatures and from tissues stored for more than one year were still immunoreactive and not degraded. Long-term studies focusing on storage time at ambient temperature are ongoing.

Limitations of the current study include the number and types of samples analyzed (20 different non-malignant and 4 different malignant tissue types), the homogenous sample size instead of including samples of different sizes, and the choice of material and techniques. Although we clearly demonstrate successful analysis of phospho-proteins from PFPE tissues in our sample collection, data for other tissue types, other sample sizes or other methods (e.g. laser microdissection) need to be obtained in future studies. Furthermore, clinical relevant situations, e.g. analysis of biopsies, have to be addressed in future prospective studies. An important strength of our study is the systematic sampling of the material in two independent laboratories (Munich and Graz) according to predefined standard operating procedures. Moreover, within the SPIDIA consortium (www.spidia.eu) five different laboratories are involved in PFPE sample collection and analysis. Thus, our results are easily transferable to other laboratories.

In summary, our data suggest that the novel formalin-free preservation technology, the PAXgene Tissue System, represents a powerful alternative to formalin for research and routine pathology applications. It preserves morphology, nucleic acids, and proteins [10], [11], [12], and as shown in the present study, phosphoproteins of clinical tissue samples in spite of pre-analytical variation such as prolonged fixation times.

## Conclusions

Besides preserving morphology and nucleic acids, the PAXgene Tissue System quantitatively conserves phosphoproteins in human tissue samples, even after prolonged fixation or stabilization times. In addition, it allows subsequent protein assays, such as 2D-PAGE and ELISA. Thus, the PAXgene Tissue System has great potential to serve as a novel multimodal fixative for modern pathology, enabling histopathological diagnosis and extensive biomarker research from the same clinical tissue specimen.

## Supporting Information

Figure S1 **The PAXgene Tissue System preserves phosphoproteins in human clinical tissue specimens.** (**A, B, C**) 12 human non-malignant (uterus, breast, prostate, bladder, spleen, gall bladder, muscle, skin, tongue, salivary gland, thyroid gland and lung) tissue specimens were each divided into three samples and either cryopreserved (cryo), fixed and stabilized in the PAXgene Tissue reagents and paraffin-embedded (PFPE) or fixed in formalin and paraffin-embedded (FFPE). Proteins were extracted with respective protocols (See Protein Extraction, Experimental Section) and 15 µg protein of each was separated by SDS-PAGE. Western blot analysis was performed using indicated antibodies.
(TIF)

Figure S2 **Proteins extracted from PFPE tissues are suitable for two-dimensional gel electrophoresis.** (**A, B, C, D**) Four human non-malignant (duodenum, colon, stomach, breast) tissue specimens were each divided into three samples each and either cryopreserved (cryo), fixed and stabilized in the PAXgene Tissue reagents and paraffin-embedded (PFPE) or fixed in formalin and paraffin-embedded (FFPE). Proteins were extracted with respective protocols (See Two-dimensional SDS-PAGE, Experimental Section) and 150 µg protein of each was separated by two-dimensional SDS-PAGE. The isoelectric focusing was conducted in a range between pH 3 – pH 10. (**A**) The duodenum sample was a replicate analysis of the same sample used in Figure 2 to evaluate reproducibility of the system.
(TIF)

Table S1 **Antibodies used for western blot analysis.**
(TIF)
