# Supplementary figures and images for: The PAXgene® Tissue System Preserves Phosphoproteins in Human Tissue Specimens and Enables Comprehensive Protein Biomarker Research
Source: PLoS One. 2013 Mar 29;8(3):e60638. doi: 10.1371/journal.pone.0060638 (PMC3612043; doi:10.1371/journal.pone.0060638)

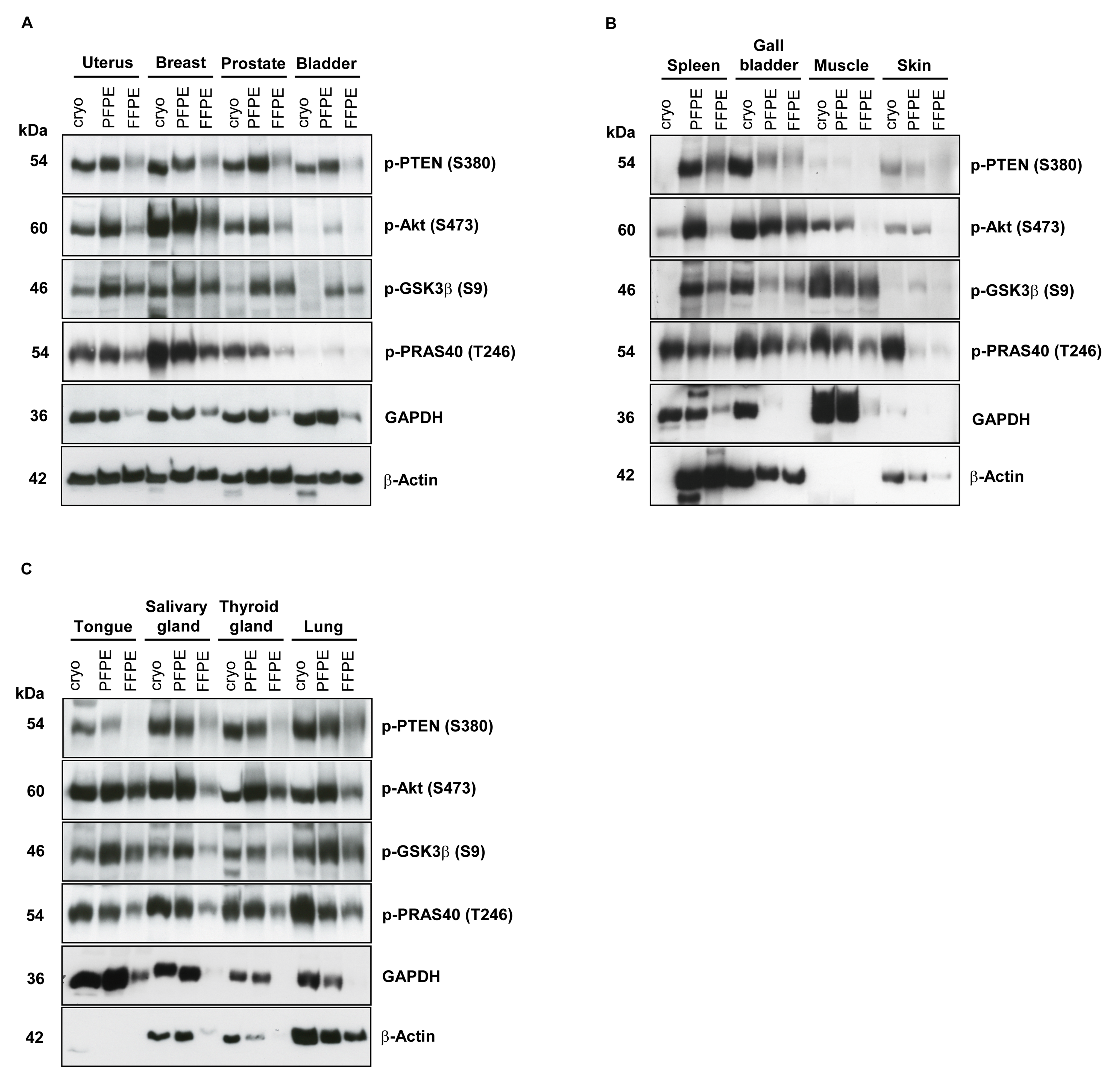

Supplement: Figure S1 — The PAXgene Tissue System preserves phosphoproteins in human clinical tissue specimens. (A, B, C) 12 human non-malignant (uterus, breast, prostate, bladder, spleen, gall bladder, muscle, skin, tongue, salivary gland, thyroid gland and lung) tissue specimens were each divided into three samples and either cryopreserved (cryo), fixed and stabilized in the PAXgene Tissue reagents and paraffin-embedded (PFPE) or fixed in formalin and paraffin-embedded (FFPE). Proteins were extracted with respective protocols (See Protein Extraction, Experimental Section) and 15 µg protein of each was separated by SDS-PAGE. Western blot analysis was performed using indicated antibodies. (TIF) [file pone.0060638.s001.tif]

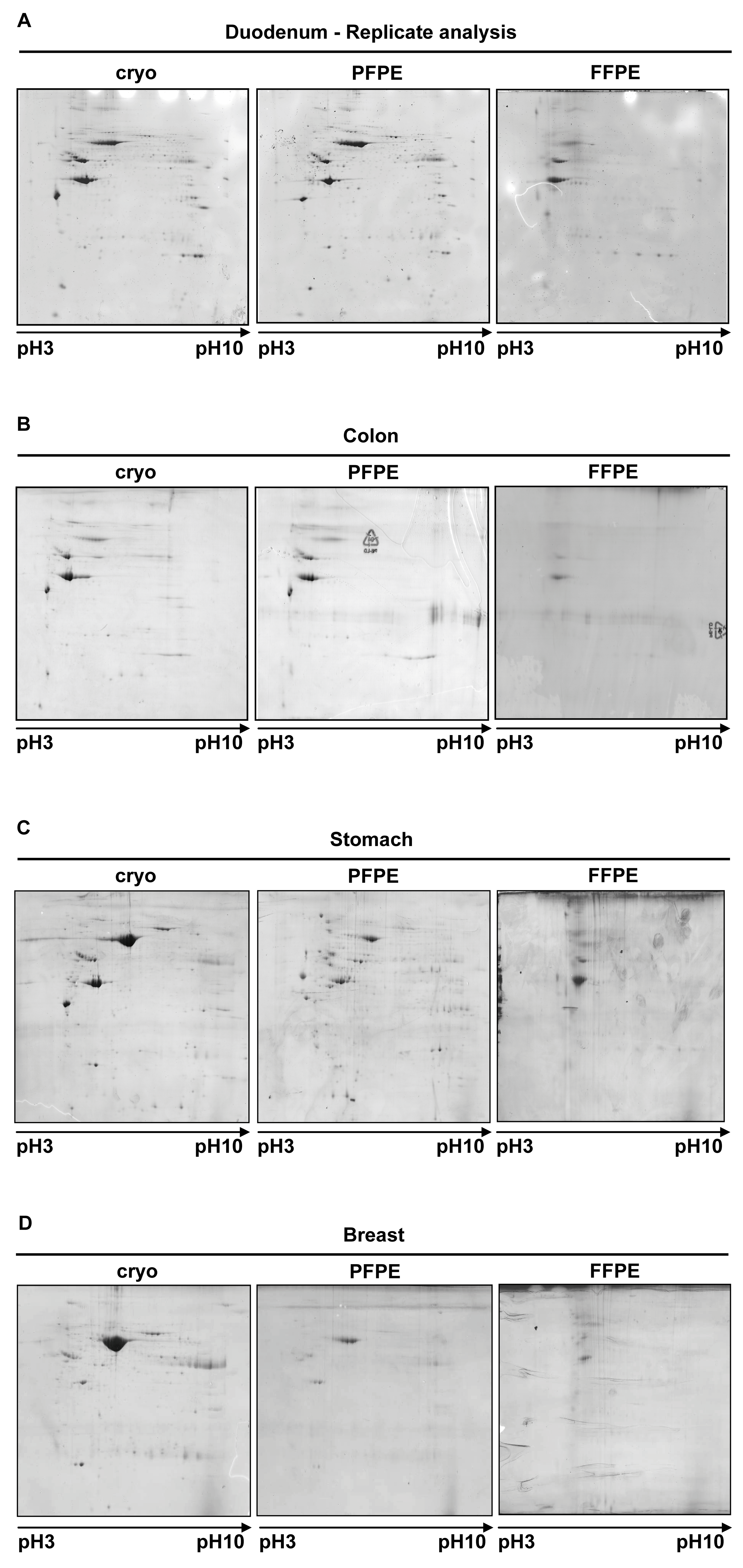

Supplement: Figure S2 — Proteins extracted from PFPE tissues are suitable for two-dimensional gel electrophoresis. (A, B, C, D) Four human non-malignant (duodenum, colon, stomach, breast) tissue specimens were each divided into three samples each and either cryopreserved (cryo), fixed and stabilized in the PAXgene Tissue reagents and paraffin-embedded (PFPE) or fixed in formalin and paraffin-embedded (FFPE). Proteins were extracted with respective protocols (See Two-dimensional SDS-PAGE, Experimental Section) and 150 µg protein of each was separated by two-dimensional SDS-PAGE. The isoelectric focusing was conducted in a range between pH 3 – pH 10. (A) The duodenum sample was a replicate analysis of the same sample used in Figure 2 to evaluate reproducibility of the system. (TIF) [file pone.0060638.s002.tif]

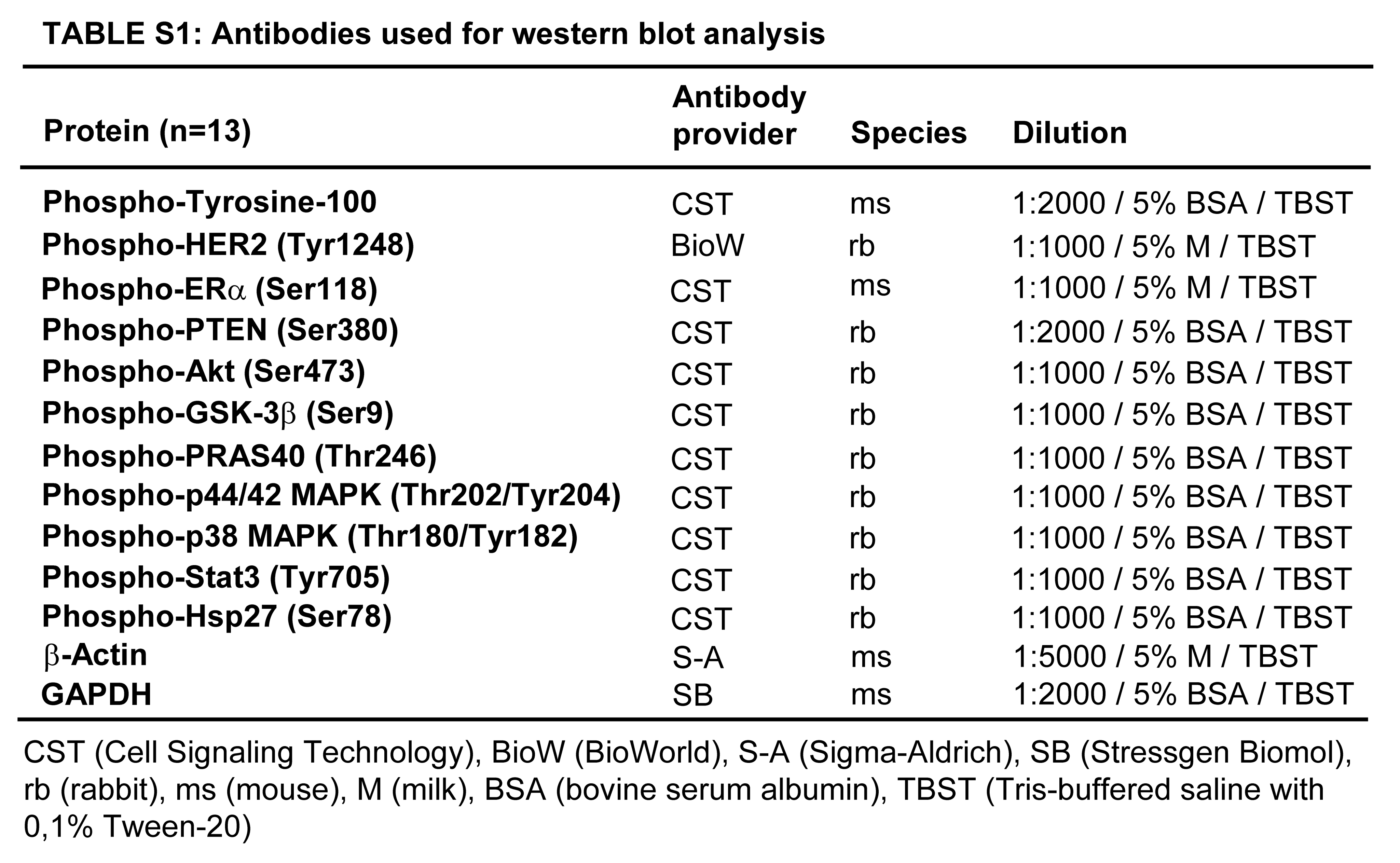

Supplement: Table S1 — Antibodies used for western blot analysis. (TIF) [file pone.0060638.s003.tif]
